# Successful transcolonic endoscopic ultrasound-guided fine-needle biopsy of retroperitoneal fibrosis

**DOI:** 10.1055/a-2518-5387

**Published:** 2025-02-11

**Authors:** Xu Ji, Zheng Zhang, Feng Du, Shutian Zhang, Peng Li

**Affiliations:** 1 26455 Department of Gastroenterology, Capital Medical University Affiliated Beijing Friendship Hospital, Beijing, China

A 54-year-old man presented to our department with a 2-month history of back pain and painful urination. A contrast-enhanced computed tomography (CT) revealed a large retroperitoneal soft tissue mass encircling the abdominal aorta, surrounding vessels, and the right ureter ( Fig. 1 ). He had an average leukocyte level (7300/µL), within the normal range for tumor markers and IgG4 levels, and a C-reactive protein level of 38.88 mg/L. The clinical picture was highly suggestive of retroperitoneal fibrosis (RPF); however, for a definite diagnosis, a biopsy of the lesion was necessary.

**Fig. 1:**
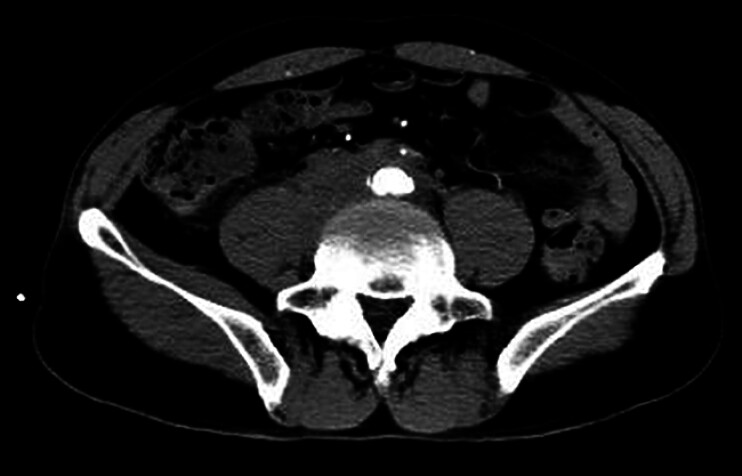
Contrast-enhanced computed tomography showed an abnormal retroperitoneal soft tissue mass encircling the abdominal aorta.

CT-guided aspiration biopsy was not an option as the blood vessels and bowel could not be avoided. Therefore, endoscopic ultrasound-guided fine-needle biopsy (EUS-FNB) was selected as the optimal method for obtaining tissue samples, as it is a minimally invasive technique that has previously demonstrated efficacy.

We decided to perform transcolonic EUS-FNB because of the lesion’s proximity to the pelvis ( Video 1 ). In addition to avoiding adjacent blood vessels, transcolonic EUS-FNB allows for a comprehensive view of the lesion from all sides. Therefore, we proceeded with an ultrasound endoscope (EG-3870UTK; Pentax, Tokyo, Japan) to the ileocecal region and then withdrew the endoscope to observe and select the optimal puncture position when the lesion was visible.

Transcolonic endoscopic ultrasound-guided fine-needle biopsy of retroperitoneal fibrosis.Video 1

EUS disclosed a diffuse echo-reduced mass surrounding the distal aorta ( Fig. 2 ), and Doppler imaging confirmed the absence of interpolated vessels. Subsequently, two locations in the ascending colon were selected for lesion puncture using a 22-gauge ultrasound biopsy needle (Cook EchoTip Procore HD Endobronchial Ultrasound Biopsy, G34281; Cook Medical, Bloomington, Indiana, USA). Biopsy results confirmed a definite histopathologic diagnosis of RPF ( Fig. 3 ), and immunohistochemical analysis indicated CD38 and Congo red staining positivity. The patient did not experience any severe adverse events, and he was successfully treated with prednisolone and azathioprine.

**Fig. 2:**
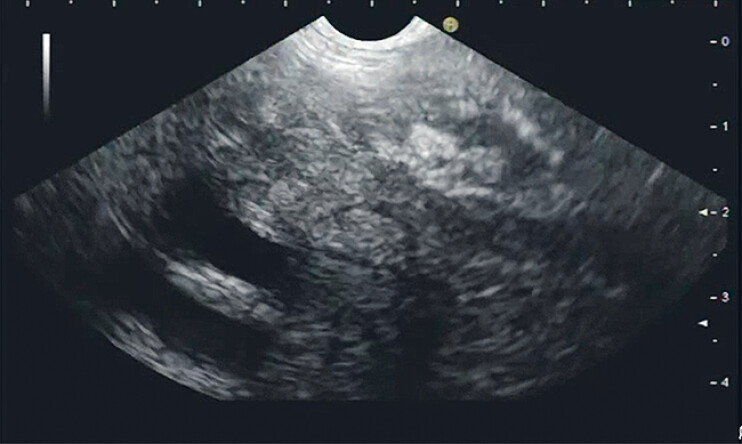
Transcolonic endoscopic ultrasound revealed a retroperitoneal, diffuse echo-reduced mass suggestive of retroperitoneal fibrosis.

**Fig. 3:**
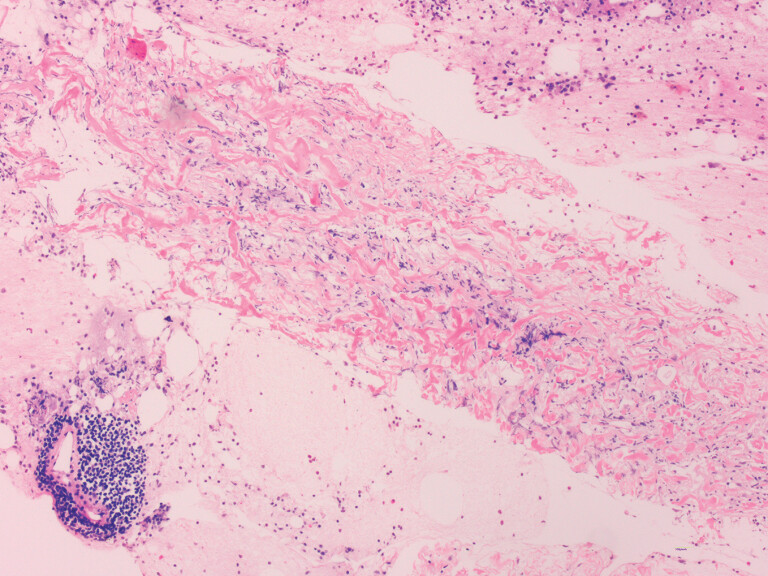
Histopathologic image of the specimen obtained from endoscopic ultrasound-guided fine-needle biopsy, showing fibrous tissue in cells, together with lymphocytes between the connective tissue fibers.

EUS-FNB can become a safe and effective diagnostic method for RPF 1 . Transduodenal EUS-FNB is unable to penetrate lesions in close proximity to the pelvic cavity. Conversely, transcolonic EUS-FNB can be utilized to identify and select multiple potential puncture sites while avoiding the vicinity of blood vessels, thereby ensuring a safe and precise procedure. Therefore, in cases of RPF affecting the lower abdomen or the region in proximity to the pelvis, a transcolonic EUS-FNB may be a viable option for diagnosis.

Endoscopy_UCTN_Code_TTT_1AS_2AZ

## Notes

Endoscopy E-Videos https://eref.thieme.de/e-videos
*E-Videos* is an open access online section of the journal *Endoscopy* , reporting on interesting cases and new techniques in gastroenterological endoscopy. All papers include a high-quality video and are published with a Creative Commons CC-BY license. Endoscopy E-Videos qualify for HINARI discounts and waivers and eligibility is automatically checked during the submission process. We grant 100% waivers to articles whose corresponding authors are based in Group A countries and 50% waivers to those who are based in Group B countries as classified by Research4Life (see: https://www.research4life.org/access/eligibility/ ). This section has its own submission website at https://mc.manuscriptcentral.com/e-videos .
